# Editorial: Modulation of the immune system by bacteria: From evasion to therapy

**DOI:** 10.3389/fimmu.2022.1112427

**Published:** 2023-01-04

**Authors:** Marina de Bernard, Maria Kaparakis-Liaskos, Mario Milco D’Elios

**Affiliations:** ^1^ Department of Biology, University of Padova, Padova, Italy; ^2^ Department of Microbiology, Anatomy, Physiology and Pharmacology, School of Agriculture, Biomedicine and Environment, La Trobe University, Melbourne, VIC, Australia; ^3^ Department of Molecular and Developmental Medicine, University of Siena, Siena, Italy

**Keywords:** bacterial mechanisms, bacteria elicited trained immunity, T cells, anti-microbial therapeutics, bacteria-based therapeutics

This Research Topic examines how bacterial pathogens avoid or inactivate host defenses in order to survive within a host. Numerous tactics are employed by bacteria, such as modulating cell surfaces, secreting proteins that inhibit or degrade host immune factors, and even mimicking host molecules to mediate pathogenesis. Knowledge of the mechanisms utilized by pathogens to mediate disease may be advantageous for developing medical treatments aimed at eliminating infection-causing bacteria from humans. Furthermore, understanding how some bacterial factors tune the immune system may facilitate the development of targeted therapies. In this editorial we provide an overview of the exciting and diverse contents of this research topic, spanning multiple aspects of microbiology and immunology.

One-half of the world’s population is colonized by *Helicobacter pylori*, a Gram-negative bacterial pathogen that is able to persist and establish chronic infection (1). The tight association of *H. pylori* with gastric cancer is established (2). Deng et al., elegantly review the effects of *H. pylori* on the microenvironment of gastric cancer, which may impair cancer immune surveillance or change the stroma of the tumor, thus promoting carcinogenesis both locally and systemically. The role of the immunomodulatory activity of *H. pylori* in favoring the onset and progression of gastric cancer (3) represents only one side of the coin. The other side is the potential application of some bacterial factors produced by the pathogen, such as the *H. pylori* neutrophil activating protein (HP-NAP), as adjuvants. This topic is extensively discussed by Codolo et al. in a review fully devoted to HP-NAP, a miniferritin with immune modulatory properties, that is becoming a promising biological therapeutic tool for the treatment of allergies and solid tumors.

The possibility that immune synapse formation between an antigen-presenting cell and a T lymphocyte might be a direct target of bacterial virulence factors is emerging as a novel means of immune evasion. Capitani and Baldari offer an overview of the evidence that has recently accumulated to support this notion.

It is established that pathogens that cause chronic inflammation promote tumorigenesis (4), but it is also true that bacteria may display tumor-targeting properties and can activate the immune system to exert anti-tumor effects (5). Decades have passed since *Bacille Calmette-Guérin* (BCG), an attenuated strain of *Mycobacterium bovis*, has been approved by the FDA as a treatment for bladder cancer. However, recently, there has been a substantial increase in the number of studies focusing on the application of bacteria as cancer therapeutics. Tang et al. present an up-to-date review of the role of bacteria in anti-cancer immunity and their use in immunotherapy as carriers of therapeutic agents. The advantages of using unmodified bacteria in comparison to engineered bacteria in immunotherapy are also discussed.

*Mycobacterium tuberculosis*, the etiologic agent of tuberculosis, remains a significant global public health burden (6). Despite being developed nearly a century ago, BCG remains the only licensed vaccine against tuberculosis (7). Opportunities to leverage knowledge regarding the immunology of *M. tuberculosis* infection to improve treatments and vaccines are growing as our understanding of host responses to *M. tuberculosis* infection increases. The findings that the mycobacterial acyl carrier protein (AcpM), a key protein involved in mycolic acid production (8), is a mycobacterial effector capable of modulating macrophage functions broaden our understanding of this pathogen. AcpM upregulates miR-155-5p to prevent the activation of the transcription factor EB (TFEB), which regulates the expression of the autophagy and lysosomal genes in macrophages, and it enhances the survival of intracellular mycobacteria by preventing phagosome-lysosome fusion (Paik et al.).

The pathophysiology of brucellosis and *M. tuberculosis* infection share several characteristics. Despite the possibility of its occurrence during the treatment of *M. tuberculosis* infection, immune reconstitution inflammatory syndrome (IRIS) has never been documented in brucellosis patients. According to a case study described by Qu et al., IRIS can happen when treating *Brucella*. A persistent parasitic infection is brought on by the pathogen’s infection of macrophages and ability to elude clearance mechanism. Mitroulis et al., taking advantage of *in vitro* and *ex vivo* approaches, describe the expression pattern of genes in the immune cell population, when they first encounter *Brucella*, throughout the sickness, and following a successful cure.

Comparatively examining immune responses to nine uropathogens in bladder infection, Li et al. list the similarities and differences between them. The findings lead the authors to suggest that various microbial bladder infections should adopt matching immunomodulatory therapies, and that distinct microbial illnesses may also make use of the same immunomodulatory intervention if they share the same potent therapeutic targets.

A crucial element of innate immunity is represented by NOD-like receptors (NLR) which act as intracellular sensors for bacteria. In their discussion of the many strategies employed by bacterial pathogens to elude detection by NLRs and eventually interfere with the development of host defense, Kienes et al. highlight how bacterial infections and their products activate NLRs to induce inflammation and illness. The possibility that NLRs, which operate by recruiting and activating caspases into inflammasomes, might be subverted by bacterial factors to alleviate inflammasome-driven diseases is also discussed.

Despite the fact many Gram-negative pathogens produce outer-membrane vesicles (OMVs) that contain immunogenic cargo, the presence of immunostimulatory molecules in OMVs produced by commensal organisms has only recently been recognized. In the study of Gilmore et al., it is reported that the cargo associated with OMVs produced by the intestinal commensal *Bacteroides fragilis* can activate host innate immune receptors such as Toll-like receptors (TLR)-2, TLR4, TLR7, and nucleotide oligomerization domain (NOD)-like receptor NOD1, whereas *B. fragilis* bacteria could only activate TLR2, suggesting that *B. fragilis* OMVs may facilitate immune crosstalk at the gastrointestinal epithelial surface.

A technique called fecal microbiota transplantation (FMT) is utilized to directly modify the recipient’s gut microbiota. FDA authorized the use of FMT in 2013 for the treatment of recurrent and resistant Clostridium difficile infection and FMT therapy has been applied beyond gastrointestinal disorders to also include extra-gastrointestinal diseases ever since (9). According to the notion that the microbiota is crucial for intestinal homeostasis in all vertebrates, intestinal bacteria-free birds (IBF) exhibit lower body weights and inferior immunological, metabolic, antioxidant, and intestinal absorption capacities than bacteria-bearing birds. The transplantation of fecal bacteria of birds from the control group into the intestines of IBF birds reshapes the intestinal immune function and metabolism (Li et al.).

Immune checkpoint inhibitors (ICIs) have been used to treat a variety of malignancies, and the results have been astounding (10). The most popular ICIs are antibodies that target the programmed cell death protein 1 (PD-1). These ICIs operate by preventing the interaction between the PD-1 receptor on T cells and the PD-L1 ligand on tumor cells, which allows T cells to detect and destroy tumor cells (11). Most colorectal cancer (CRC) patients do not react to anti-PD-1 therapy because the tumor microenvironment lacks sufficient tumor-infiltrating lymphocytes (Bai et al.). Using a mouse model of CRC, it was demonstrated that treatment with FMT plus anti-PD-1 antibodies improved survival and tumor control in mice compared to treatment with anti-PD-1 therapy or FMT alone (Huang et al.).

An updated viewpoint on autotransporter (AT) proteins, the central part of a molecular nano-machine that transports cargo proteins through the outer membrane of Gram-negative bacteria, is provided by Clarke et al. By expanding the knowledge of the connections between structure and function of ATs, the study gives insights into the variety of ATs that may direct future research aimed at addressing several open questions about autotransporters.

In order to shed light on the protective mechanism underpinning vibriosis resistance in fish, Zhou et al. employed genomic, transcriptomic, and experimental methods. This work provides essential genetic resources for breeding and controlling infectious diseases in fish culture.

Hormones may modulate host responses to pathogens and dysmetabolic conditions. It has been recently reported that in obese patients chronic low-grade inflammation is driven by the CD300e antigen (12). Brettle et al. elegantly review the interactions between sex hormones, gut microbiome, and intestinal inflammation in obesity. The epidemiology, etiology, and outcomes of obesity and its associated metabolic problems clearly exhibit sexual dimorphisms, with females frequently experiencing more protection than males. This defense has mostly been credited to variations in fat distribution and the female sex hormone estrogen. More recently, changes in gut microbiota and intestinal immune system have also been linked to the sexual dimorphisms of obesity.

Males were generally more susceptible to *Nocardia* infection and disease than females. However, Han et al. by investigating the interplay between estradiol and immune response to *Nocardia*, demonstrated increased severity in *Nocardia*-infected female mice compared to male mice with increased mortality, elevated lung bacterial loads, and an exaggerated pulmonary inflammatory response that was mimicked in ovariectomized female mice supplemented with 17β-estradiol. Authors underlines the importance to include and separately evaluate both sexes in the future research on *Nocardia* immune responses.

Collectively, the wide-ranging studies and reviews presented in this research topic highlight the multiple mechanisms whereby bacterial pathogens promote disease and reveal novel insights and targets to combat bacterial infections and bacterial-mediated pathologies. On the other hand, the amount of evidence supporting the use of bacterial-derived bioactive materials for therapeutic purposes has been steadily increasing. Accordingly, the present collection includes critical findings on the great potential of bacterial organisms and their active components in the biomedical field, especially in cancer therapy.

## Author contributions

MdB, MK-L, MMDE: Prepared and discussed about this Research Topic, invited authors, revised their manuscripts, and handled their revisions. All authors contributed to the article and approved the submitted version.
